# Evaluation of Abdominal Computed Tomography Scans for Differentiating the Discrepancies in Abdominal Adipose Tissue Between Two Major Subtypes of Primary Aldosteronism

**DOI:** 10.3389/fendo.2021.647184

**Published:** 2021-07-16

**Authors:** Kuan-Ming Chen, Bo-Ching Lee, Po-Ting Chen, Kao-Lang Liu, Kuan-Heng Lin, Chin-Chen Chang, Tung-Hsin Wu, Jia-Sheng Hong, Yen-Hung Lin

**Affiliations:** ^1^ Department of Biomedical Imaging and Radiological Sciences, National Yang-Ming University, Taipei, Taiwan; ^2^ Department of Biomedical Imaging and Radiological Sciences, National Yang Ming Chiao Tung University, Hsinchu, Taiwan; ^3^ Industrial Ph.D. Program of Biomedical Science and Engineering, School of Biomedical Science and Engineering, National Yang-Ming University, Taipei, Taiwan; ^4^ Industrial Ph.D. Program of Biomedical Science and Engineering, School of Biomedical Science and Engineering, National Yang Ming Chiao Tung University, Hsinchu, Taiwan; ^5^ Department of Medical Imaging, National Taiwan University Hospital and National Taiwan University College of Medicine, Taipei, Taiwan; ^6^ Department and Graduate Institute of Forensic Medicine, National Taiwan University College of Medicine, Taipei, Taiwan; ^7^ Department of Internal Medicine, National Taiwan University Hospital and National Taiwan University College of Medicine, Taipei, Taiwan

**Keywords:** primary aldosteronism, abdominal computed tomography, idiopathic hyperaldosteronism, aldosterone-producing adenoma, abdominal adiposity indexes

## Abstract

The aim of this study was to analyze the differences in the distribution of abdominal adipose tissue between the two subtypes of primary aldosteronism (PA) using abdominal computed tomography. We retrospectively analyzed patients diagnosed as having essential hypertension (EH) or PA from the prospectively collected Taiwan Primary Aldosteronism Investigation (TAIPAI) database. Patients with PA were divided into the subgroups of idiopathic hyperaldosteronism (IHA) and unilateral aldosterone-producing adenoma (APA). Patients’ basic clinicodemographic data were collected, and a self-developed CT-based software program was used to quantify the abdominal adiposity indexes, including visceral adipose tissue (VAT) area, VAT ratio, waist circumference (WC), subcutaneous adipose tissue (SAT) area, and SAT ratio. We included 190 patients with EH and 436 patients with PA (238 with IHA and 198 with APA). The APA group had significantly lower abdominal adiposity indexes than the other groups. We also found negative correlations of aldosterone-to-renin ratio (ARR) with VAT area, VAT ratio, WC, and body mass index (BMI) in the APA group. After propensity score matching (which left 184 patients each in the IHA and APA groups), patients in the APA group still had significantly lower WC, SAT area, SAT ratio, and VAT ratio than those in the IHA group. Furthermore, logistic regression analysis indicated that lower probability of abdominal obesity was significantly related to patients with APA. Our data revealed that the distribution of abdominal adipose tissue was similar in patients with IHA and those with EH, but the abdominal adiposity indexes were significantly lower in patients with APA than in those with IHA and EH.

## Introduction

PA is one of the most common types of endocrine hypertension, with a prevalence of 5%–13% in patients with hypertension (1, 2). It is characterized by high plasma aldosterone concentration (PAC) and low plasma renin activity (PRA). PA has been considered a rare disease, but recent studies have determined that up to 20% of patients with resistant hypertension have PA (3–5).

Abdominal obesity is a common risk factor for cardiovascular events in patients with hypertension. Patients with EH have a similar probability of developing lipid metabolism disorder to those with PA (6, 7). In addition, an increased PAC may unstable the metabolic complications associated with abdominal adiposity, leading to a high risk of morbidity and mortality (6, 8–10). This implies that the etiology for developing the metabolic syndromes of obesity, dyslipidemia, and hyperglycemia in patients with PA might differ from that in patients with EH (11, 12).

Among patients with PA, 64% have IHA caused by bilateral adrenal hyperplasia and 27% have APA (13). Moreover, two subtypes of PA (IHA and APA) have different pathogeneses, and patients with APA have much higher levels of aldosterone than do those with IHA (14, 15). Therefore, patients with APA should theoretically experience severer obesity-related disorders than those with IHA due to aldosterone excess. However, studies have reported that patients with IHA have more metabolic disorders and a higher prevalence of obesity than those with APA (16, 17). We accordingly assumed that the distribution and mechanisms of the development of abdominal obesity in the two subtypes of PA should also be different. Therefore, using patients with EH as the control group, we investigated the relationship between abdominal fat tissue distribution and pathophysiology, and the factors affecting them, in the two subtypes of PA.

## Materials and Methods

### Case Collection

This was a retrospective analysis of a prospectively collected database. We enrolled patients diagnosed as having EH and PA from the Taiwan Primary Aldosteronism Investigation (TAIPAI) database from 2010 to 2018. We collected patients’ abdominal computed tomography (CT) scans and basic clinicodemographic data, including sex, age, BMI, systolic blood pressure (SBP), diastolic blood pressure (DBP), potassium ion concentration, PAC, PRA, ARR, and estimated glomerular filtration rate (eGFR). Patients’ history of hypertension and type 2 diabetes (T2D) were also collected. All patient identifying information was removed from the CT scans before analysis, and the requirement to obtain informed consent was waived by the institutional review board.

### Classification of PA Subtypes

PA was diagnosed based on the following biochemical criteria: (a) autonomous excess aldosterone production evidenced with an ARR > 35; (b) a TAIPAI score > 60% (18); and (c) post-saline loading PAC > 10 ng/dL, ARR > 35 in a post-captopril/losartan test, or PAC > 6 ng/dL indicated by a fludrocortisone suppression test.

The classification of PA into subtypes was based on TAIAPI experience (2, 19). IHA was diagnosed according to the following criteria: (a) evidence of bilateral diffuse enlargement on preoperative computed tomography (CT); (b) nonlateralization of aldosterone secretion during adrenal venous sampling; and (c) diffuse cell hyperplasia on biopsy of resected specimen in patients who underwent an operation. APA was diagnosed based on the following criteria: (a) biochemical finding of PA and the evidence of adenoma on preoperative CT; (b) lateralization of aldosterone secretion during adrenal venous sampling; and (c) pathologically proven adenoma after adrenalectomy and (d) the subsequent emergence of either a cure pattern of hypertension without antihypertensive agents and improvement in hypertension, potassium, PAC, and PRA (14).

### Quantification of Abdominal Adipose Tissue Using Multidetector CT

**Figure 1:**
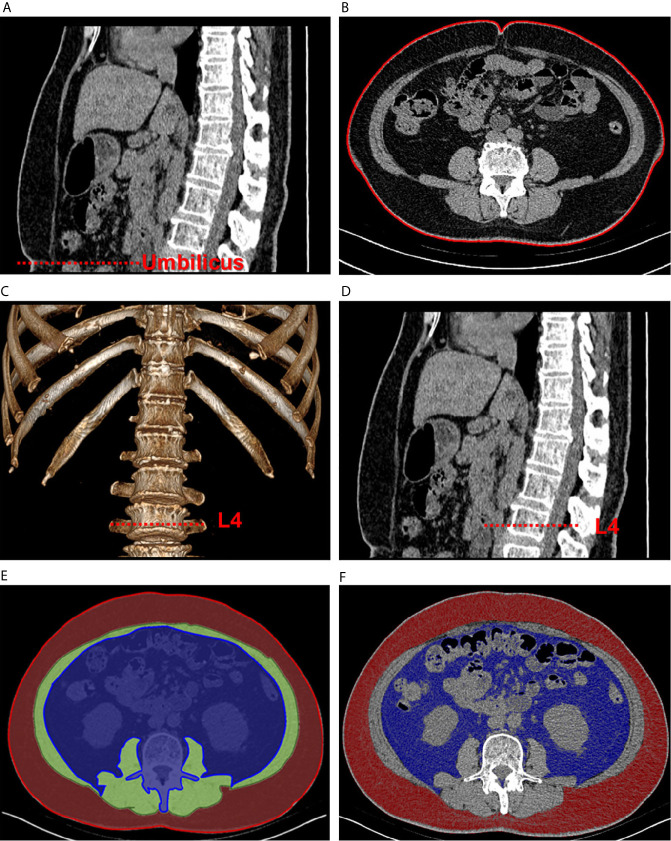
Schematic of abdominal adipose tissue quantification using computed tomographic images. The sagittal plane **(A)** was used to locate the umbilical area, and one of the corresponding transverse planes **(B)** was selected to measure waist circumference. Then, volume rendering **(C)** was used to locate the L4 area **(D)**. The regions of interest of visceral fat (blue), muscular layer (green), and subcutaneous fat (red) were circled in one of the transverse planes **(E)**. Quantification of the fat areas was completed through threshold segmentation **(F)**.

CT was used to calculate WC and quantify the parameters of abdominal adipose tissue. The CT scanning parameters were as follows: the tube voltage was 120 kVp, and the tube current was under automated exposure control. Slice thickness was 5 mm, and the scan range went from the upper edge of T12 to S1. A self-developed program written on the MATLAB platform was used to measure abdominal adipose tissue and its relevant indexes. To set the threshold segmentation, the Hounsfield unit (HU) values were set between −190 and −30 for fat and between −29 and 150 for muscle (20). The procedure is depicted in **Figure 1**. First, a slice of the umbilical area was selected (**Figure 1A**) to measure WC (**Figure 1B**). Next, one of the slices at L4 transverse process was selected (**Figures 1C, D**). The regions of interest in the layer of VAT, layer of muscle, and layer of SAT were circled on the slice to calculate the area of the total abdomen (**Figure 1E**). Finally, the set HU values for fat was used for threshold segmentation, and the corresponding SAT and VAT areas were calculated (**Figure 1F**). To eliminate individual differences, the SAT and VAT areas were divided by the total abdomen area to ensure standardized SAT and VAT ratios.

### Statistical Analysis

Statistical analysis was performed using SPSS v22.0 (IBM Corp., Armonk, NY, USA). Data were presented as mean ± standard deviation for continuous variables and as percentage for categorical variables. Nonnormally distributed variables, such as PAC, PRA, and ARR, were expressed as median and interquartile range. Significant difference were compared using one-way ANOVA with the Bonferroni post-hoc for continuous variables, Chi-square test for categorical variables and Kruskal–Wallis tests for nonnormally distributed variables among EH, IHA, and APA groups or between any two groups. Spearman’s rank correlation was calculated for each of the three groups, and linear correlations among BMI, abdominal adiposity indexes, and ARR were analyzed.

A 1:1 propensity score matching (PSM) analysis was conducted. Propensity scores were estimated to control for possible confounding bias, such as sex, age, and BMI between any two groups in this study. The independent samples t test was used to compare continuous variables between two groups. We also performed univariate and multivariate logistic regression analysis to detect the relationship between the two subtypes of PA, clinical data and abdominal adiposity indexes.

## Results

### EH *vs*. PA

We included 190 patients diagnosed with EH and 436 diagnosed with PA. The EH group had a higher proportion of men than the PA group did, but age and BMI were not significantly different between the two groups. The PA group exhibited significantly higher SBP, higher DBP, and longer duration of hypertension compared with the EH group. Significantly higher PAC, higher ARR, lower PRA, and lower potassium ion concentration were observed in the PA group compared with the EH group. Analysis of abdominal adiposity indexes indicated that the PA group had significantly lower WC, total abdomen area, VAT area, and VAT ratio than the EH group. The results of before and after PSM for sex between EH and PA groups are presented in **Table S1**.

### EH *vs*. Two Subtypes of PA

In total, 238 patients with IHA and 198 patients with APA who were scheduled to undergo adrenalectomy. **Table 1** compares the clinical and demographic variables between the groups. The EH group had a significantly higher proportion of men than the IHA group did, but not compared with the APA group. SBP and DBP were higher in the IHA and APA groups than in the EH group, but the duration of hypertension was significantly longer in patients with IHA than in those with EH. Compared with the EH group, the APA group had significantly higher ARR and both the APA and IHA groups had significantly higher PAC, higher ARR, lower PRA, and lower potassium ion concentration. Analysis of abdominal adiposity indexes indicated that the SAT ratio was significantly higher in the IHA group than in the EH group. However, the EH and IHA groups did not differ significantly in terms of WC, total abdomen area, SAT area, VAT area, and VAT ratio. By contrast, the APA group had significantly lower WC, total abdomen area, SAT area, VAT area, and VAT ratio than the EH group did. The results of PSM for sex, age, and BMI for the EH group *vs*. the IHA group and the EH group *vs*. the APA group are presented in **Table S2**.

**Table 1:** Comparison of clinicodemographic data and abdominal adiposity indexes among the EH, IHA, and APA groups.

Variables	EH	IHA	APA	Overall *p*-value	*p*-value between each two groups		
	(n = 190)	(n = 238)	(n = 198)		EH *vs*. IHA	EH *vs*. APA	IHA *vs*. APA
Clinicodemographic data							
Sex, male (%)^(a)^	115 (61%)	110 (46%)	101 (51%)	*<0.05*	*<0.05*	*0.18*	*0.95*
Age, years	54.56 ± 14.85	54.39 ± 11.10	51.29 ± 10.79	*<0.05*	*1.00*	*<0.05*	*<0.05*
BMI, kg/m^2^	25.93 ± 4.90	26.03 ± 3.89	24.95 ± 4.11	*<0.05*	*1.00*	*0.08*	*<0.05*
Duration of hypertension, years	5.41 ± 7.92	7.39 ± 8.41	6.56 ± 6.33	*<0.05*	*<0.05*	*0.42*	*0.79*
Presence of type 2 diabetes (%)^(a)^	25 (13%)	45 (19%)	34 (17%)	*0.26*	*0.33*	*0.87*	*1.00*
SBP, mmHg	146.76 ± 26.29	153.39 ± 18.96	153.78 ± 20.80	*<0.01*	*<0.01*	*<0.01*	*1.00*
DBP, mmHg	86.77 ± 16.49	92.96 ± 13.01	92.71 ± 13.96	*<0.001*	*<0.001*	*<0.001*	*1.00*
Potassium, mmol/L	4.11 ± 0.44	3.83 ± 0.56	3.49 ± 0.61	*<0.001*	*<0.001*	*<0.001*	*<0.001*
PAC^(b)^, ng/dL	31.48	42.20	45.09	*<0.001*	*<0.05*	*<0.001*	*<0.05*
	(22.27 to 46.63)	(31.50 to 61.95)	(30.78 to 74.80)				
PRA^(b)^, ng/mL/h	1.67	0.31	0.23	*<0.001*	*<0.001*	*<0.001*	*0.67*
	(0.33 to 4.95)	(0.10 to 0.62)	(0.10 to 0.56)				
ARR^(b)^	20.95	153.46	233.60	*<0.001*	*0.14*	*<0.001*	*<0.001*
	(9.62 to 99.77)	(70.34 to 422.84)	(70.13 to 640.50)				
eGFR, mL/min/1.73m^2^	85.96 ± 25.12	92.73 ± 32.11	88.80 ± 25.86	*0.11*	*0.16*	*1.00*	*0.47*
Abdominal adiposity indexes							
WC, cm	84.61 ± 10.26	84.17 ± 9.80	80.52 ± 9.84	*<0.001*	*1.00*	*<0.001*	*<0.001*
Total abdomen area, cm^2^	624.36 ± 166.42	606.30 ± 146.13	575.39 ± 157.99	*<0.01*	*0.70*	*<0.01*	*0.12*
SAT area, cm^2^	172.04 ± 79.00	178.77 ± 71.29	150.21 ± 63.06	*<0.001*	*0.99*	*<0.01*	*<0.001*
VAT area, cm^2^	162.85 ± 85.38	156.04 ± 72.06	131.54 ± 82.89	*<0.001*	*1.00*	*<0.001*	*<0.01*
SAT ratio	0.27 ± 0.07	0.29 ± 0.08	0.25 ± 0.07	*<0.001*	*<0.01*	*0.42*	*<0.001*
VAT ratio	0.24 ± 0.08	0.24 ± 0.07	0.21 ± 0.08	*<0.001*	*1.00*	*<0.001*	*<0.001*

Data were presented as mean ± SD, median (interquartile range), or number (%). EH, essential hypertension; IHA, idiopathic hyperaldosteronism; APA, aldosterone-producing adenoma; BMI, body mass index; SBP, systolic blood pressure; DBP, diastolic blood pressure; PAC, plasma aldosterone concentration; PRA, plasma renin activity; ARR, aldosterone–renin ratio; WC, waist circumference; SAT area, area of subcutaneous adipose tissue; VAT area, area of visceral adipose tissue; SAT ratio was calculated by dividing SAT area by total abdomen area; VAT ratio was calculated by dividing VAT area by total abdomen area. ^(a)^chi-square test; ^(b)^Kruskal-Wallis test.

### Relationships of ARR With Factors

**Figure 2:**
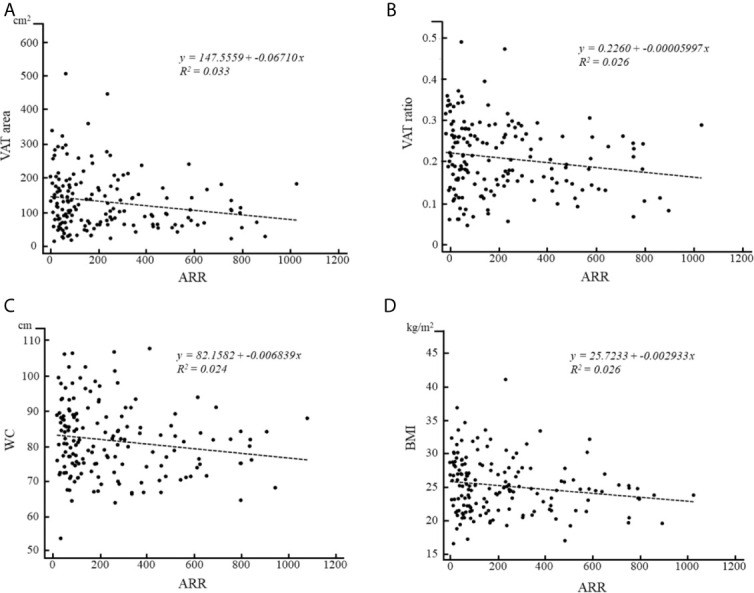
Correlations of ARR with **(A)** VAT area, **(B)** VAT ratio, **(C)** WC and **(D)** BMI in patients with APA.

No significant correlations were observed between ARR, abdominal adiposity indexes, and BMI in the PA group. However, in the APA group, negative correlations of ARR were identified with VAT area (r = −0.174, p < 0.05; **Figure 2A**), VAT ratio (r = −0.177, p < 0.05; **Figure 2B**), WC (r = −0.182, p < 0.05; **Figure 2C**), and BMI (r = −0.176, p < 0.05; **Figure 2D**); however, no statically significant correlations were observed with total abdomen area (r = −0.152, p = 0.06), SAT area (r = −0.132, p = 0.09), or SAT ratio (r = −0.038, p = 0.63). By contrast, in the patients with EH or IHA, no statically significant correlations were observed of ARR with any factors.

### IHA *vs*. APA

As shown in **Table 1**, age and BMI were significantly higher in the IHA group than in the APA group. PAC and ARR were significantly higher and potassium ion concentration was significantly lower in patients with APA than in those with IHA. Analysis of abdominal adiposity indexes indicated that the APA group had significantly lower WC, SAT area, VAT area, SAT ratio, and VAT ratio than the other groups.

**Figure 3:**
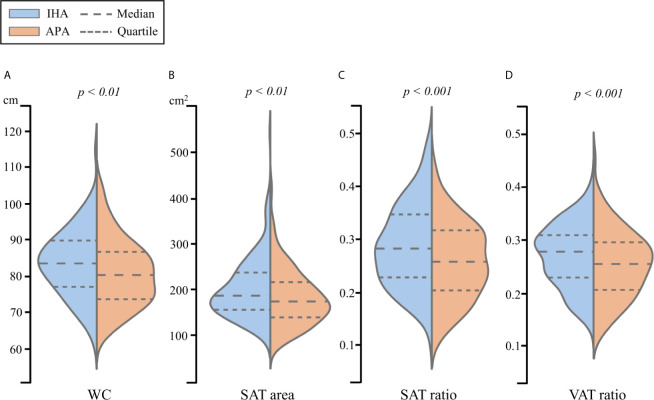
The violin plot of **(A)** WC, **(B)** SAT area, **(C)** SAT ratio and **(D)** VAT ratio for propensity score matched patients between IHA and APA.

The clinical data and abdominal adiposity indexes after PSM for age and BMI, which led to 184 patients each in IHA and APA groups, are listed in **Table 2**. No significant differences in sex, duration of hypertension, presence of T2D, SBP, DBP, PAC, or PRA were noted. However, as was the case before PSM, ARR remained significantly higher and potassium ion concentration remained significantly lower in patients with APA than in those with IHA. Furthermore, the APA group had significantly lower values of all the abdominal adiposity indexes than the IHA group, including WC, SAT area, SAT ratio and VAT ratio (**Figure 3**).

**Table 2:** Comparison of clinicodemographic data and abdominal adiposity indexes between the IHA and APA groups after propensity score matching for age and BMI.

Variables	IHA	APA	*p*-value
	(n = 184)	(n = 184)	
Clinicodemographic data			
Sex, male (%)^(a)^	96 (52%)	89 (47%)	*0.39*
Age, years	53.35 ± 11.06	52.66 ± 9.72	*0.52*
BMI, kg/m^2^	25.96 ± 3.83	25.21 ± 4.08	*0.07*
Duration of hypertension, years	7.08 ± 8.41	6.87 ± 6.43	*0.79*
Presence of type 2 diabetes (%)^(a)^	28 (15%)	33 (18%)	*0.31*
SBP, mmHg	152.32 ± 18.65	153.89 ± 20.56	*0.44*
DBP, mmHg	92.60 ± 12.36	92.22 ± 13.53	*0.77*
Potassium, mmol/L	3.82 ± 0.54	3.51 ± 0.61	*<0.001*
PAC^(b)^, ng/dL	42.600	45.500	*0.14*
	(31.90 to 62.60)	(30.55 to 74.72)	
PRA^(b)^, ng/mL/h	0.310	0.220	*0.18*
	(0.10 to 0.62)	(0.097 to 0.52)	
ARR^(b)^	155.750	235.380	*<0.01*
	(67.81 to 455.86)	(70.34 to 620.35)	
eGFR, mL/min/1.73m^2^	93.84 ± 33.91	87.21 ± 24.23	*<0.05*
Abdominal adiposity indexes			
WC, cm	84.22 ± 9.51	81.39 ± 9.48	*<0.01*
Total abdomen area, cm^2^	597.32 ± 140.91	586.31 ± 157.00	*0.48*
SAT area, cm^2^	173.62 ± 68.27	153.61 ± 63.28	*<0.01*
VAT area, cm^2^	151.73 ± 70.52	137.09 ± 83.04	*0.06*
SAT ratio	0.58 ± 0.15	0.52 ± 0.14	*<0.001*
VAT ratio	0.24 ± 0.07	0.21 ± 0.08	*<0.001*

Data were presented as mean ± SD, median (interquartile range), or number (%). IHA, idiopathic hyperaldosteronism; APA, aldosterone-producing adenoma; BMI, body mass index; SBP, systolic blood pressure; DBP, diastolic blood pressure; PAC, plasma aldosterone concentration; PRA, plasma renin activity; ARR, aldosterone–renin ratio; WC, waist circumference; SAT area, area of subcutaneous adipose tissue; VAT area, area of visceral adipose tissue; SAT ratio was calculated by dividing SAT area by total abdomen area; VAT ratio was calculated by dividing VAT area by total abdomen area. ^(a)^chi-square test; ^(b)^Kruskal-Wallis test.

### Univariate and Multivariate Logistic Regression Analysis Between IHA and APA

Logistic regression for WC, SAT ratio, VAT ratio, potassium ion concentration, ARR and eGFR were performed to distinguish APA from IHA after PSM for age and BMI. As shown in **Table 3**, our multivariate regression analysis data revealed that patients with APA exhibited lower SAT ratio, VAT ratio and potassium ion concentration than patients with IHA. We additionally provide regression data before executing PSM in **Table S3**, the results also show that patients with APA has a lower chance of developing abdominal obesity and more severe hypokalemia, and are irrelevant to age and BMI.

**Table 3:** Logistic regression analysis of WC, SAT ratio, VAT ratio, potassium concentration, ARR and eGFR between IHA and APA group after propensity score matching for age and BMI.

Variables	Univariate Regression Analysis				Multivariate Regression Analysis			
	*β*	OR	95% CI of OR	*p*-value	*β*	OR	95% CI of OR	*p*-value
WC, cm	-0.032	0.969	0.948 – 0.991	*<0.05*	-0.004	0.996	0.966 - 1.027	*0.793*
SAT ratio	-5.138	0.006	0.000 – 0.096	*<0.01*	-4.559	0.010	0.000 - 0.336	*<0.05*
VAT ratio	-3.834	0.022	0.002 – 0.289	*<0.05*	-5.271	0.005	0.000 - 0.216	*<0.01*
Potassium, mmol/L	-0.930	0.394	0.269 – 0.579	*<0.001*	-0.828	0.437	0.290 - 0.658	*<0.001*
ARR	0.001	1.000	1.000 – 1.000	*<0.01*	0.001	1.000	1.000 - 1.000	*<0.05*
eGFR, mL/min/1.73m^2^	-0.008	0.992	0.985 – 0.999	*<0.05*	-0.008	0.992	0.984 - 1.001	*0.073*

IHA, idiopathic hyperaldosteronism; APA, aldosterone-producing adenoma; β, Coefficient of regression equation; OR, Odds ratio; CI, Confidence interval; WC, waist circumference; SAT, subcutaneous adipose tissue; VAT, visceral adipose tissue; SAT ratio was calculated by dividing SAT area by total abdomen area; VAT ratio was calculated by dividing VAT area by total abdomen area; ARR, aldosterone–renin ratio. For the OR, IHA group was coded as 0, APA group was coded as 1.

## Discussion

This study is unique in that it involves 436 sets of abdominal CT scans and clinical data from patients with PA; in contrast to other studies, we added an additional 190 sets of data from patients with EH for comparison. The strength of our study is the direct use of CT scans to assess the abdominal component, in particular, the SAT and VAT can be measured directly and quantified using the following indexes, area and ratio. Our approach should be more convincing than other previous studies that use weight, BMI, or WC for assessment (16, 17). Our study reveals that the APA group had significantly lower abdominal adiposity indexes than the other groups. We also found negative correlations of ARR with VAT area, VAT ratio, WC, and BMI in the APA group. After PSM, patients in the APA group still had significantly lower WC, SAT area, SAT ratio, and VAT ratio than those in the IHA group. Furthermore, the logistic regression analysis indicated that lower probability of abdominal obesity was significantly related to patients with APA.

Adipose tissue is involved in many physiological and pathological processes. Excessive adipose tissue often causes with excessive PAC, causing the mineralocorticoids continuously activated and strengthened. This finally leads to the inflammation and adipocyte differentiation (21–24). Studies have indicated that the prevalence of obesity or dyslipidemia does not differ significantly between EH and PA groups (6, 25). Although our results indicated that the PA group had significantly lower WC and VAT than the EH group did, these differences disappeared after PSM for sex (**Table S1**).

However, PSM for sex, age, and BMI yielded no significant differences in WC or other abdominal adiposity indexes between patients with EH and IHA, whereas patients with APA had significantly lower WC, total abdomen area, SAT and VAT than those with EH (**Table S2**). This demonstrates that the metabolic phenotypes of obesity are similar in the EH and IHA groups, which is consistent with previous research (7). The present data suggested that, in contrast to patients with IHA, the metabolic profile of patients with APA might be a crucial factor that lowers the obesity prevalence in the overall PA population.

Distribution of abdominal fat is an important predictor of cardiovascular risk, increasing accumulation of SAT and VAT are associated with several cardiac diseases, particularly the VAT is an independent factor for metabolic syndrome (MetS) in patients with obesity (26). Whereas, interesting was that recent studies had indicated that patients with APA had higher risk of developing cardiac events among the PA population (27, 28). And despite having lower PAC than patients with APA, only those with IHA are more likely to develop MetS (16, 17).

Our study at least provides another point of view to support these inconsistent issues. In this study, patients with IHA and APA exhibited different distributions of abdominal adipose tissue. Even after controlling for age and BMI, patients with APA exhibited significant lower WC, SAT and VAT than patients with IHA. According to our logistic regression analysis, patients with IHA and APA exhibited significant differences in SAT and VAT. Consequently, it can be assumed that the higher incidence of cardiac events in the APAs is not associate with obesity, but may be direct related to the toxicity of chronic excess aldosterone.

ARR is an indicator of disorder in aldosterone activity and is useful for the diagnosis of PA (29). In particular, ARR is highly sensitive and specific in diagnosing APA (30, 31). In the present study, ARR was significantly negatively correlated with VAT area, VAT ratio, WC, and BMI in patients with APA. These findings were consistent with Er et al., who indicated that patients with APA had relatively smaller abdominal fat distribution, but the VAT will be increased after unilateral adrenalectomy (32). However, relevant study is still sparse, and the exact pathomechanisms remain clarified in the future.

No relationship was observed between ARR and other factors in patients with IHA. By contrast, Shibayama et al. (33) had reported that PAC was positively proportional to visceral fat distribution in patients with IHA. Taken together, the findings indicate that the relationship of abdominal obesity with aldosterone activity may differ between IHA and APA. These findings are in line with the notion that long-term aldosterone excess in patients with APA may lead to inflammation and fibrosis of perirenal fat tissue, eventually resulting in reduced abdominal adiposity indexes (34).

This retrospective study had the following limitations. First, we did not exclude autonomous cortisol secretion in patients with APA. Cortisol hypersecretion can be associated with obesity. Second, we did not evaluate laboratory data, such as lipid profile and HOMA-IR index, but these data have been proven to be irrelevant to abdominal fat accumulation (32). Third, this retrospective study did not take the effects of relevant medication history into account, such as antihypertensives or statins.

In conclusion, our data revealed that the abdominal adipose tissue indexes were similar in patients with IHA and those with EH but were markedly lower in patients with APA. Furthermore, ARR was negatively correlated with VAT area, VAT ratio, WC, and BMI in patients with APA but not in patients with IHA. These findings provide new insights into the relationship between adipose tissue and aldosterone excess in patients with APA and IHA. This suggests that patients with APA could be relatively lean during clinical practice, but still need to beware of higher risk of cardiac disease, such as heart failure, atrial fibrillation, ischemic heart disease, and other vascular events.

## Data Availability Statement

The original contributions presented in the study are included in the article/**Supplementary Material**. Further inquiries can be directed to the corresponding authors.

## Ethics Statement

The studies involving human participants were reviewed and approved by National Taiwan University Hospital Research Ethics Committee. The patients/participants provided their written informed consent to participate in this study.

## Author Contributions

K-MC and J-SH carried out the experiment. K-MC wrote the manuscript with support from B-CL, P-TC, and K-HL. K-LL and Y-HL conceived the original idea. C-CC and T-HW supervised the project. All authors contributed to the article and approved the submitted version.

## Funding

This study was supported by the research grants MOST109-2314-B-010 -023 -MY3 from the Ministry of Science and Technology, Taipei, Taiwan to T-HW.

## Conflict of Interest

The authors declare that the research was conducted in the absence of any commercial or financial relationships that could be construed as a potential conflict of interest.
