# Supplementary material for: Evaluation of Abdominal Computed Tomography Scans for Differentiating the Discrepancies in Abdominal Adipose Tissue Between Two Major Subtypes of Primary Aldosteronism
Source: Front Endocrinol (Lausanne). 2021 Jul 16;12:647184. doi: 10.3389/fendo.2021.647184 (PMC8323492; doi:10.3389/fendo.2021.647184)
Supplement: Supplementary file 1 [file Table_1.docx]

**Table S1** Comparison of clinical data and abdominal adiposity indexes between the EH and PA groups before and after propensity score matching.

|  | Before propensity score matching | | | After propensity score matching^(c)^ | | |
| --- | --- | --- | --- | --- | --- | --- |
| Variables | EH  (n=190) | PA  (n=436) | *p*-value | EH  (n=190) | PA  (n=190) | *p*-value |
| Clinicodemographic data | | | | | | |
| Sex, male (%)^(a)^ | 115 (61%) | 211 (48%) | *<0.05* | 115 (61%) | 115 (61%) | *0.91* |
| Age, years | 54.56 ±14.85 | 52.98 ± 11.05 | *0.14* | 54.56 ±14.86 | 54.31 ±10.92 | *0.84* |
| BMI, kg/m^2^ | 25.93 ± 4.90 | 25.54 ± 4.02 | *0.31* | 25.93 ±4.90 | 26.22 ± 3.69 | *0.52* |
| Duration of hypertension, years | 5.41 ± 7.92 | 7.01 ± 7.54 | *<0.05* | 5.41 ±7.93 | 7.57 ±8.50 | *<0.05* |
| Presence of type 2 diabetes (%)^(a)^ | 25 (13%) | 79 (18%) | *0.15* | 25 (13%) | 33 (17%) | *0.31* |
| SBP, mmHg | 146.76 ± 26.29 | 153.57 ± 19.80 | *<0.001* | 146.77 ±26.29 | 153.37 ±19.86 | *<0.01* |
| DBP, mmHg | 86.77 ± 16.49 | 92.84 ± 13.43 | *<0.001* | 86.78 ± 16.50 | 93.44 ±13.38 | *<0.001* |
| Potassium, mmol/L | 4.11 ± 0.44 | 3.68 ± 0.60 | *<0.001* | 4.14 ± 0.53 | 3.80 ± 0.56 |  |
| PAC^(b)^, ng/dL | 31.48  (22.27 to 46.63) | 42.90 (31.27 to 65.10) | *<0.001* | 31.480  (22.27 to 46.63) | 39.640  (31.50 to 62.11) | *<0.001* |
| PRA^(b)^, ng/mL/h | 1.67 (0.33 to 4.95) | 0.27 (0.10 to 0.60) | *<0.001* | 1.670  (0.33 to 4.95) | 0.305  (0.10 to 0.62) | *<0.001* |
| ARR^(b)^ | 20.95 (9.62 to 99.77) | 175.71 (70.31 to 517.77) | *<0.001* | 20.950  (9.62 to 99.77) | 156.170  (65.32 to 443.70) | *<0.001* |
| eGFR, mL/min/1.73m^2^ | 85.96 ± 25.12 | 90.91 ± 29.44 | *0.15* | 87.40 ±25.00 | 90.52 ±34.10 | *0.31* |
| Abdominal adiposity indexes | | | | | | |
| WC, cm | 84.61 ± 10.26 | 82.51 ± 9.97 | *<0.05* | 84.61 ±10.26 | 85.20 ±9.15 | *0.55* |
| Total abdomen area, cm^2^ | 624.36 ± 166.42 | 592.26 ± 152.24 | *<0.05* | 624.36 ± 166.42 | 615.52 ±144.01 | *0.58* |
| SAT area, cm^2^ | 172.04 ± 79.00 | 165.80 ± 69.09 | *0.32* | 172.05 ±79.00 | 172.37 ±67.23 | *0.96* |
| VAT area, cm^2^ | 162.85 ± 85.38 | 144.91 ± 78.04 | *<0.05* | 162.85 ± 85.38 | 162.93 ±72.08 | *0.99* |
| SAT ratio | 0.27 ± 0.07 | 0.26 ± 0.07 | *0.33* | 0.27 ±0.08 | 0.28 ±0.07 | *0.27* |
| VAT ratio | 0.24 ± 0.08 | 0.23 ± 0.08 | *<0.05* | 0.24 ± 0.08 | 0.26 ±0.08 | *0.44* |

Data were presented as mean ± SD, median (interquartile range), or number (%)

EH, essential hypertension; BMI, body mass index; DM, diabetes mellitus; SBP, systolic blood pressure; DBP, diastolic blood pressure; PAC, plasma aldosterone concentration; PRA, plasma renin activity; ARR, aldosterone–renin ratio; WC, waist circumference; SAT area, area of subcutaneous adipose tissue; VAT area, area of visceral adipose tissue; SAT ratio was calculated by dividing SAT area by total abdomen area; VAT ratio was calculated by dividing VAT area by total abdomen area. ^(a)^ chi-square test; ^(b)^ Kruskal-Wallis test; ^(c)^1:1 Matching for sex
