# Supplementary material for: Evaluation of Abdominal Computed Tomography Scans for Differentiating the Discrepancies in Abdominal Adipose Tissue Between Two Major Subtypes of Primary Aldosteronism
Source: Front Endocrinol (Lausanne). 2021 Jul 16;12:647184. doi: 10.3389/fendo.2021.647184 (PMC8323492; doi:10.3389/fendo.2021.647184)
Supplement: Supplementary file 2 [file Table_2.docx]

**Table S2** Comparison of clinicodemographic data and abdominal adiposity indexes between the EH and IHA groups and between the EH and APA groups after propensity score matching.

| Variables | EH  (n=187) | IHA  (n=187) | *p*-value | EH  (n=181) | APA  (n=181) | *p*-value |
| --- | --- | --- | --- | --- | --- | --- |
| Clinicodemographic data | | | | | | |
| Sex, male (%)^(a)^ | 112 (59%) | 108 (57%) | *0.75* | 108 (59%) | 99 (57%) | *0.39* |
| Age, years | 54.78 ± 14.83 | 54.36 ± 11.17 | *0.76* | 53.97 ± 14.45 | 51.96 ± 10.67 | *0.13* |
| BMI, kg/m^2^ | 25.86 ± 4.88 | 26.21 ± 3.94 | *0.44* | 25.75 ± 4.61 | 25.14 ± 4.162 | *0.19* |
| Duration of hypertension, years | 5.48 ± 7.97 | 7.70 ± 8.66 | *< 0.05* | 5.36 ± 7.971 | 6.84 ± 6.378 | *0.06* |
| Presence of type 2 diabetes (%)^(a)^ | 24 (12%) | 36 (19%) | *0.12* | 23 (12%) | 32 (17%) | *0.24* |
| SBP, mmHg | 146.84 ± 26.42 | 153.80 ± 19.76 | *< 0.01* | 147.31 ± 24.45 | 154.70 ± 20.69 | *< 0.01* |
| DBP, mmHg | 86.83 ± 16.57 | 93.04 ±13.52 | *< 0.001* | 87.18 ± 15.30 | 92.92 ± 14.15 | *< 0.001* |
| Potassium, mmol/L | 4.12 ± 0.51 | 3.80 ± 0.55 | *< 0.001* | 4.14 ± 0.52 | 3.50 ± 0.61 | *< 0.001* |
| PAC^(b)^, ng/dL | 31.480 (22.34 to 46.80) | 39.640 (31.55 to 61.80) | *< 0.01* | 31.92  (22.25 to 47.09) | 44.69  (30.350 to 74.72) | *< 0.001* |
| PRA^(b)^, ng/mL/h | 1.560 (0.33 to 4.89) | 0.310 (0.10 to 0.60) | *< 0.001* | 1.61  (0.32 to 4.95) | 0.24  (0.100 to 0.553) | *< 0.001* |
| ARR^(b)^ | 21.220 (10.04 to 101.07) | 155.750 (72.16 to 407.19) | *< 0.01* | 21.08  (9.62 to 103.17) | 228.32  (67.65 to 601.70) | *< 0.001* |
| eGFR, mL/min/1.73m^2^ | 87.50 ± 25.19 | 89.29 ± 31.94 | *0.54* | 88.26 ± 24.55 | 88.07 ± 24.54 | *0.94* |
| Abdominal adiposity indexes | | | | | | |
| WC, cm | 84.39 ± 10.15 | 84.90 ± 9.94 | *0.61* | 84.26 ± 10.18 | 81.10 ± 9.95 | *< 0.01* |
| Total abdomen area, cm^2^ | 621.03 ± 164.66 | 619.42 ± 149.12 | *0.92* | 620.62 ± 165.98 | 583.74 ± 161.72 | *< 0.05* |
| SAT area, cm^2^ | 170.84 ± 77.85 | 176.74 ± 73.89 | *0.45* | 170.68 ± 76.49 | 151.01 ± 64.73 | *< 0.01* |
| VAT area, cm^2^ | 162.24 ± 85.80 | 161.92 ± 73.03 | *0.96* | 160.32 ± 86.14 | 134.60 ± 85.57 | *< 0.01* |
| SAT ratio | 0.27 ± 0.07 | 0.28 ± 0.07 | *0.13* | 0.27 ± 0.07 | 0.25 ± 0.07 | *0.07* |
| VAT ratio | 0.24 ± 0.08 | 0.25 ± 0.07 | *0.73* | 0.24 ± 0.07 | 0.21 ± 0.08 | *< 0.001* |

Data were presented as mean ± SD, median (interquartile range), or number (%).

^(a)^ chi-square test; ^(b)^ Kruskal-Wallis test
