# Supplementary material for: Evaluation of Abdominal Computed Tomography Scans for Differentiating the Discrepancies in Abdominal Adipose Tissue Between Two Major Subtypes of Primary Aldosteronism
Source: Front Endocrinol (Lausanne). 2021 Jul 16;12:647184. doi: 10.3389/fendo.2021.647184 (PMC8323492; doi:10.3389/fendo.2021.647184)
Supplement: Supplementary file 3 [file Table_3.docx]

**Table S3** Logistic regression analysis of WC, SAT ratio, VAT ratio, age, BMI, potassium concentration and ARR between IHA and APA group before propensity score matching.

| Variables | Univariate Regression Analysis | | | | Multivariate Regression Analysis | | | |
| --- | --- | --- | --- | --- | --- | --- | --- | --- |
|  | *β* | OR | 95% CI of OR | *p*-value | *β* | OR | 95% CI of OR | *p*-value |
| WC, cm | -0.038 | 0.963 | 0.944 – 0.982 | *< 0.001* | -0.003 | 0.997 | 0.961 – 1.034 | *0.868* |
| SAT ratio | -6.031 | 0.002 | 0.000 – 0.034 | *< 0.001* | -7.215 | 0.001 | 0.000 – 0.018 | *< 0.001* |
| VAT ratio | -0.544 | 0.004 | 0.000 – 0.050 | *< 0.001* | -5.416 | 0.004 | 0.000 – 0.180 | *< 0.01* |
| Age, years | -0.026 | 0.974 | 0.958 – 0.992 | *< 0.01* | -0.021 | 0.979 | 0.958 – 1.001 | *0.058* |
| BMI, kg/m^2^ | -0.068 | 0.934 | 0.890 – 0.981 | *< 0.01* | 0.003 | 1.003 | 0.924 – 1.088 | *0.940* |
| Potassium, mmol/L | -0.993 | 0.371 | 0.261 – 0.526 | *< 0.001* | -0.836 | 0.433 | 0.295 – 0.636 | *<0.01* |
| PAC, ng/dL | 0.006 | 1.006 | 1.001 – 1.012 | *< 0.05* | 0.001 | 1.001 | 0.995 – 1.007 | *0.672* |
| ARR | 0.001 | 1.000 | 1.000 – 1.000 | *< 0.01* | 0.001 | 1.000 | 1.000 – 1.000 | *<0.01* |

IHA, idiopathic hyperaldosteronism; APA, aldosterone-producing adenoma; *β*, Coefficient of regression equation; OR, Odds ratio; CI, Confidence interval; WC, waist circumference; SAT, subcutaneous adipose tissue; VAT, visceral adipose tissue; SAT ratio was calculated by dividing SAT area by total abdomen area; VAT ratio was calculated by dividing VAT area by total abdomen area; ARR, aldosterone–renin ratio. For the OR, IHA group was coded as 0, APA group was coded as 1.
